# The link between mental health and safe drinking water behaviors in a vulnerable population in rural Malawi

**DOI:** 10.1186/s40359-019-0320-1

**Published:** 2019-07-08

**Authors:** Jurgita Slekiene, Hans-Joachim Mosler

**Affiliations:** EAWAG, Swiss Federal Institute of Aquatic Science and Technology, Environmental Social Sciences, Environmental and Health Psychology, Überlandstrasse 133, P.O. Box 611, CH-8600 Dübendorf, Switzerland

**Keywords:** Behavior change, Rural Malawi, RANAS, Mental health, Public health, Water collection, Transportation and storage

## Abstract

**Background:**

Mental disorders, particularly depression and post-traumatic stress disorder, are common long-term psychological outcomes in emergency contexts arising from conflicts, natural disasters, and other challenging environmental conditions. In emergencies, people suffer not only from the lack of external resources such as drinking water and food but also from poor mental health. Mental disorders can substantially impair daily activities in vulnerable individuals. However, water, sanitation, and hygiene (WASH) behaviors are daily activities that require effort, time, and strong internal motivation. Therefore, questions arise: whether there is a relationship between mental health and safe water behaviors, and if so, whether the motivational drivers of these behaviors are affected by mental health.

**Methods:**

Our cross-sectional study conducted face-to-face interviews with 638 households in rural Malawi. We used a quantitative questionnaire based on the risks, attitudes, norms, abilities, and self-regulation (RANAS) approach to measure motivational psychosocial factors. Mental health was assessed using the validated Chichewa version of the Self-Reporting Questionnaire (SRQ-20). Results. Almost a third of the respondents reported poor mental health. We found significant negative association between mental health and self-reported safe water collection (*p* = .01, *r* = −.104) but not between safe water transportation and storage behavior. The moderation analysis revealed significant interaction effects of mental health with some psychosocial factors and therefore on WASH behaviors. Poor mental health changed the influence of three psychosocial factors—perceived others’ behavior, commitment, and remembering—on safe drinking water collection behavior. The influence on water transportation and storage behavior of the perceived severity of contracting a disease, the belief that transporting and storing water requires substantial effort, and others’ approval depended on the mental health condition of the respondent.

**Conclusions:**

These results imply that populations with a significant proportion of individuals with poor mental health will benefit from interventions to mitigate mental health before or parallel to behavioral change interventions for WASH. Specific population-level interventions have been shown to have a positive effect on mental well-being, and they have been successfully applied at scale. This research is especially relevant in emergency contexts, as it indicates that mental health measures before any WASH interventions will make them more effective.

## Background

Water is a fundamental human right, but around 783 million people worldwide still have no access to safe drinking water [1]. Many international organizations and local governments in developing countries try to make drinking water available for vulnerable populations by constructing and maintaining protected water sources such as boreholes and by treating water to make it safe to drink.

However, simply providing infrastructure such as boreholes does not always result in safe drinking water collection, transportation, and storage [2]. Contamination of drinking water can occur at several stages between water source and point of use, such as while transporting and storing drinking water [3–5]. Collecting drinking water from safe water sources and transporting and storing it safely requires specific behaviors, and substantial behavior change interventions are often required before these are generally and regularly performed [6–9].

Moreover, water collection, transportation, and storage are daily activities that require effort, time, and self-efficacy. However, there is evidence that internal mental conditions such as poor mental health and depression can substantially impair such daily activities in vulnerable individuals [10]. Daily activities such as safe drinking water collection, transportation, and storage behaviors may be adversely influenced by mental health. More than 300 million people worldwide (3.4% of the global population) are affected by depression and other mental disorders, and their prevalence is especially high among vulnerable populations living in poverty [11] and with insecure access to water distribution systems [12, 13]. Therefore, whether there is a direct and/or indirect association or link between mental health and safe water collection, transportation, and storage behaviors is a particularly salient question.

Malawi is a particularly suitable environment in which to examine these effects. The prevalence of mental disorders in Malawi is 29.9% [14] and of depression around 30.3% [15]. Studies from Malawi report associations between depression and poverty, relationship difficulties, HIV infection, infant health problems [16], lower perceived social support, and intimate partner violence [17]. Evidence suggests that mental health may be adversely affected by insecure access to key resources such as safe water, by food insecurity and experiencing hunger in daily life [18, 19], as a consequence of iron deficiency and anemia [20], by chronic health problems, and by individuals exposed to humanitarian emergencies, natural disasters, conflicts, and other kinds of violence or abuse [21]. In vulnerable populations, it is common that people suffer from poor physical and mental health, and this has negative consequences for health-related behaviors. One recent study from Zimbabwe has shown the negative influence of depression on hand washing in children [22].

Our study identifies the effects of mental health on factors associated with water collection, transportation, and storage behavior in the study population. The aim of our research is to design effective evidence-based interventions focusing on water collection, transportation, and storage in the households of rural Malawi that take the effects of mental health into account.

**Fig. 1:**
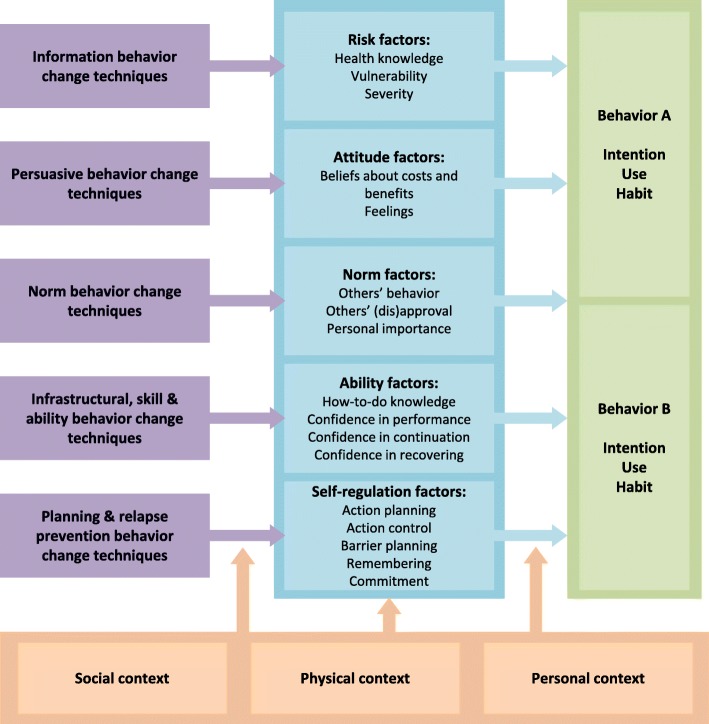
RANAS Model [23, 24]

To identify the factors associated with safe drinking water behavior, we used the risks, attitudes, norms, abilities, and self-regulation (RANAS) approach to behavior change presented in Fig. 1 [23]. The RANAS model offers an extensively tested instrument for the identification of behavior factors in the public health and water, sanitation, and hygiene (WASH) sector. The applicability of the RANAS approach to safe water behavior change has been amply demonstrated in previous research, for instance in rural Ethiopia [8], rural Benin [25], and Chad [26, 27].

The RANAS model uses five blocks of factors. Risk factors include health knowledge about transmission of a disease, prevention options, personal consequences, perceived vulnerability, and the perceived severity of contracting a disease. Attitude factors include beliefs about the costs and benefits of a particular behavior and feelings associated with the behavior. Norm factors, such as the perceived behavior of others’, others’ approval, and personal importance all involve perceived social influence. Ability factors include people’s confidence in their performance of a particular behavior. Self-regulation factors include management of conflicting goals, distracting cues and barriers, commitment, and remembering to perform the behavior. Additionally, the RANAS model provides three categories of context factors: the social, the physical, and the personal. Culture, social relations, laws and policies, economic conditions, and the information environment constitute the social context. The natural and built environments comprise the physical context. Age, gender, education, individual differences in the physical and mental health of the person, and specific condition such as experiencing hunger are included in the personal context.

The influence of psychosocial factors on the desired behavior may be impaired by context factors. Research studies have suggested that context factors such as burden of disease, access to water, household and community sanitation facilities, sociodemographic factors, and income are significant predictors for water collection, transportation, and storage behaviors [28, 29]. In this paper, we focus on an aspect of the personal context: individual differences in mental health.

We assume that impaired mental health has a negative direct and indirect influence on safe drinking water collection, transportation, and storage behavior. We therefore addressed these four research questions (RQs): 1) Which psychosocial factors are behavioral determinants a) for the safe drinking water collection and b) for water transportation and storage? 2) Is there a relationship between mental health and a) safe drinking water collection and b) water transportation and storage? 3) Does mental health moderate a) safe drinking water collection behavior and b) water transportation and storage? 4) Are there differences between individuals with good and poor mental health in RANAS psychosocial factors influencing safe drinking water collection behavior and b) water transportation and storage?

## Methods

### Study design

The study included 638 randomly selected households in rural Malawi. A cross-sectional study design was applied. The large number of study households resulted in sample statistical power for the analysis. According to Cohen [30] an alpha level of .05 and small population effect size for ANOVA calculations requires a sample size of 393 respondents when comparing two groups.

### Research area

The study took place in a rural area in Malawi, Kasungu district, in the traditional authority (TA) of Kapelula. Face-to-face household interviews and observations were conducted in five group villages in the Kapelula region, chosen by random sampling: Chikgang’ombe, Kapelula, Chinyanga, Chimwaye, and Msulira.

### Data collection method and data collector training

Quantitative data were gathered from 638 respondents using the random-route sampling method (every third household). The quantitative data collection was conducted in May and June 2017 using tablet devices equipped with OpenDataKit software (ODK). A team of 16 data collectors performed structured face-to-face household interviews and observed the availability of a specific container with lid for safe drinking water transportation and storage at one point in time during a home visit. Researchers, the Kasungu district Red Cross officers, and the field supervisor of the data collection team coordinated and monitored the sampling and interview procedure throughout the two-week period of quantitative data collection.

Prior to data collection, the data collectors attended five days of training, during which they learned about the research study, its goals, the theoretical background of the questionnaire, and the questionnaire itself. The data collectors practiced how to ask the different types of questions and how to use the questionnaire on the tablet device. The last day of training was used for a pre-test (*N =* 16) of the questionnaire to verify its applicability. Every data collector practiced an interview with a household. Field issues and prior interview experience were discussed as the final training topic.

### Questionnaires and measures

The structured, face-to-face interviews were conducted in Chichewa, the local language of the Kapelula region. The questionnaire was designed using the psychosocial factors from the RANAS model, but other questions and measurements were added. Most of the questions were closed, such as those about the target behaviors and the psychosocial factors (see also example items in Table 1). The quantitative questionnaire, based on the RANAS model, covered demographic and context questions, health status and awareness, safe drinking water collection, transportation, and storage behaviors, and psychosocial factors underlying safe drinking water collection behaviors. Questions were measured on 5-point Likert scale [from ‘not at all’ to ‘very much’; from ‘at no time’ to ‘almost each time’; from ‘never’ to ‘very often’; from ‘nobody’ to ‘almost all of them’]. Demographics (contextual factors) included gender, age in years, marital status, education in years, literacy, household size, income, wealth index, experiencing of hunger, anxiety about the future situation of the family, and diarrhea (scales and questions see in Table 1). In addition, ownership of specific container with lid for safe drinking water transportation and storage was observed and recorded.

**Table 1:** Questionnaire on the RANAS psychosocial factors (e.g., factors and items for safe water behaviors), water related behaviors, and contextual factors

Behavior Determinants	Selected Items
Risk Factors	
Vulnerability	In general, how high do you think is the risk that you get diarrhea?
Severity	Imagine that you contracted diarrhea how severe would be the impact on your life in general?
Health Knowledge	Can you tell me what causes diarrhea? Could you please tell me for each following aspects whether it is a cause or not? E.g. Water contaminated by bacteria.
Attitudinal Factors	
Belief effort	How effortful do you think is collecting drinking water from safe well?
Belief time consuming	How time consuming do you think it is to always collect drinking water from safe well?
Belief expensive	How expensive is it for you to always transport and storage water in a specific water container with lid?
Belief distance (far away)	Do you think that the safe well of drinking water is far away from your usual area of activity?
Belief certain for prevention	How certain are you that always drinking water from safe well prevents you and your family from getting diarrhea?
Feelings	How much do you like collecting drinking water from safe well?
Normative Factors	
Others’ behavior household	How many people of your household always collect drinking water from safe well?
Others’ behavior village	How many people of your village always collect drinking water from safe well?
Others’ approval	People who are important to you like your family members, friends, the chief of the village, NGO workers or Pastor, how much they approve that you always collect drinking water from safe well?
Personal obligation	How strong do you feel a personal obligation to yourself to always collect drinking water from safe well?
Ability Factors	
Confidence in performance	How sure are you that you can always collect drinking water from safe well?
Difficult water	How difficult is to get as much drinking water as you need from safe well?
Barriers distance	How confident are you that you can have drinking water from safe well, even if you have to walk some distance to reach the next safe well?
Self-Regulation Factors	
Coping plan	Do you have a plan what to do so that you always have drinking water from a safe well? Plan, please specify.
Remembering (pay attention)	How much do you pay attention to collecting drinking water from safe well?
Remembering (forgetting last 24 h)	When you think about the last 24 h: How often did it happen that you forgot to collect drinking water from safe well?
Commitment (important)	How important is it for you to collect drinking water from safe well?
Commitment (committed)	How committed do you feel to collect drinking water from safe well?
Behavior	
Water collection behavior	How often do you collect drinking water from safe well?
Water transportation & storage behavior	How often do you transport and storage water in a specific water container with lid? [Only owners of a specific water container with lid were assessed]
Contextual factors	
Wealth index	Five items: ownership of radio, TV, mobile phone, electricity, and running water yes/no; sum scale from min. 0 to max. 5
Hunger	Do you suffer from hunger often? 5-point Likert scale from 1 – never to 5 – very often
Anxiety about the future situation of the family	How anxious are you about the future situation of your family? 5-point Likert scale from 1 – not at all to 5 – very much
Diarrhea	How frequently do you suffer from diarrhea? 6-point rating scale from 1 - never to 6 - more than one day per week

Notes. Response scales: 5-point Likert scale for all RANAS psychosocial factors and behaviors [from ‘not at all’ to ‘very much’; from ‘at no time’ to ‘almost each time’; from ‘never’ to ‘very often’; from ‘nobody’ to ‘almost all of them’], [yes; no; I don’t know]. Health knowledge sum scale ranged from min. 0 to max. 20 (yes/no questions)

For our study, behavior measures included self-reported drinking water collection from a safe well and self-reported drinking water transportation in specific containers with lids (see Table 1). Only owners of a specific container with lid were asked the question about drinking water transportation and storage in a specific container with lid.

To identify hidden behavior mechanisms in our study population, we included a specific questionnaire on mental health. Mental health was assessed using the validated Chichewa version of the Self-Reporting Questionnaire (SRQ-20), which includes 20 Yes/No questions [31]. The suggested cutoff point for an initial validation study was a score of ≥7 (score range 0–20) [32]. We defined a binary variable for good and poor mental health based on this score. People who scored equal or above 7 points were assigned to a poor mental health group, and people who scored less than 7 points were assigned to a good mental health group [32].

### Statistical analysis of data

Statistical analysis of data was performed with the IBM SPSS 23 Statistics software and Microsoft Excel. Frequencies, correlations, ANOVAs, t-tests, and regression analyses were calculated. For regression analysis, we used (1) safe water collection and (2) transportation and storage behaviors as outcomes (the dependent variables) and the psychosocial factors of the RANAS model as predictors (the independent variables). A regression analysis method, PROCESS (macro for SPSS 23) [33], was used for calculations of moderation models. Such models test for interaction when two variables influence each other’s effects. Our model used mental health as a moderator (M), water collection behaviors as outcomes (Y), and psychosocial factors from the RANAS model as predictors (X). All the psychosocial factors included in the regression analyses were tested separately within a statistical moderations model as predictors (X). Moderation was conducted by testing for interaction between moderator M (mental health) and predictors X (psychosocial factors) in a model with outcome Y (water collection behavior). With evidence that X’s effect is moderated by M, the analysis should confirm X’s effect on Y at various values of the moderator (1 = poor vs 0 = good mental health in our model).

## Results

The majority of the household respondents (59.2%; *N =* 378) were women, usually the primary caregivers of their families. The rest of the study participants (40.8%; *N =* 260) were men. The age of the participants ranged from 16 to 92 years (*M* = 38.51; *SD* = 15.40). In our sample, 69% (*N =* 440) of the participants reported that they could read and write. On average, five people lived in a household (*SD* = 2.22). The average monthly income per household was 11.482 (*SD* = 22160) Malawian Kwacha (approx. 16 USD).

### The prevalence of mental disorders in rural Malawi

From the sample of 638 respondents in households of rural Malawi, around 26.8% (*N =* 171) scored equal to or above 7 on the SRQ-20 scale (*M =* 4.46, *SD =* 3.99, SRQ-20 cutoff point ≥7). More than a quarter of the respondents reported poor mental health. Of 171 respondents with poor mental health, 63.2% (*N =* 108) were female and 36.8% (*N =* 63) were male.

### The characteristics of people with poor mental health

To identify the characteristics of people with poor mental health, we compared two groups, those with poor mental health and those with good mental health, concerning these contextual factors: gender, age in years, marital status, education in years, literacy, household size, income, wealth index (ownership of radio, TV, mobile phone, electricity, and running water, answered with yes/no; measured on a sum scale from min. 0 to max. 5), experiencing of hunger, anxiety about the future situation of the family, and diarrhea.

The ANOVA mean comparison analysis of contextual factors (Table 2) revealed that individuals with poor mental health experience significantly more hunger, are more anxious about the future situation of the family, and suffer more from diarrhea. Further analysis (Chi-square) showed no gender differences between people with poor mental health and those with good mental health but significant differences in marital status and literacy.

**Table 2:** Mean comparison with ANOVA of contextual factors of the study participants on mental health condition: poor versus good

Variables	Scale	Good mental health M (SD) and %	Poor mental health M (SD) and %
Gender	Male/Female	female 57.8%	female 63.2%
Age in years		38.39 (15.29)	38.83 (15.73)
Marital status***	Yes/No (married = 1, others = 0)	married 87.7%	married 70.6%
Educ. in years		5.97 (3.57)	5.58 (3.76)
Literacy**	Yes/No	Yes 72.4%	Yes 59.6%
Household size		5.46 (2.28)	5.25 (2.21)
Income (MWK: Malawi Kwacha)		12296.00	9273.73
Wealth Index (radio, TV, mobile phone, electricity, running water)	Yes/No; sum scale range: min. 0 to max. 5	.95 (1.02)	.87 (1.00)
Hunger***	Likert 5-point scale from 1 to 5	2.60 (1.52)	3.18 (1.39)
Anxiety about health situation***	Likert 5-point scale from 1 to 5	1.78 (1.22)	2.25 (1.38)
Diarrhea**	Likert 6-point scale from 1 to 6	1.42 (.67)	1.62 (.90)

Note. *p ≤ .05, **p ≤ .01, ***p ≤ .001. Good mental health *N* = 467; poor mental health *N* = 171. Questions: Do you suffer from hunger often? Measure ranged from 1 – never to 5 – very often. How anxious are you about the future situation of your family? How frequently do you suffer from diarrhea? Response: from 1 - never to 6 - more than one day per week

### Behavior frequencies of safe water collection, transportation, and storage

Our frequencies analysis revealed that our respondents collected drinking water from a safe well on average ‘most of the times’ (*M =* 3.97, *SD =* 1.54; self-reported; *N* = 621). Observations showed that around 30.1% of the respondents (*N* = 172) stored drinking water in specific containers with lids. Only those owners of a specific container with lid were asked about drinking water transportation and storage in a specific container with lid (*N =* 172). The owners of specific containers with lids reported on average that they transport and storage drinking water in a specific container with a lid ‘most of the times’ (*M =* 4.27, *SD =* 1.22; see Table 3).

**Table 3:** Means (M) and standard deviations (SD) of safe water collection, transportation, and storage behavior

Behavior	N	M (SD)
Drinking water collection from safe well (self-reported)	621	3.97 (1.54)
Drinking water transportation and storage in a specific container with lid (self-reported)	172 (only owners of a container with lid)	4.27 (1.22)

Note. Questions for safe water collection: How often do you collect drinking water from safe well? Observation for water storage: Can you show me a water container for water collection? Water transportation question: How often do you transport and storage water in a specific water container with lid? Measure ranged from 1 – I (almost) never do this to 5 – (almost) each time

### RQ1: which psychosocial factors are behavioral determinants a) for the safe drinking water collection and b) for water transportation and storage?

To answer our first research question and so identify which psychosocial factors influence safe water collection behavior, we applied multiple linear regression analysis using self-reported safe drinking water collection behavior as outcome and RANAS psychosocial factors as predictors. All study participants, irrespective of their mental health condition, were included in the regression analyses. The RANAS model explained 74.6% of the variance in the safe drinking water collection behavior.

Eight factors were significant predictors of safe drinking water collection in the household sample (see Table 4): belief effort, belief distance (far away), others’ behavior household, others’ behavior village, difficult water (ability), remembering (pay attention), remembering (forgetting last 24 h), and communication. Belief effort (*β* = −.065) and belief distance (far away) (*β* = −.114) are negatively associated with safe water transportation behavior; if people perceive that safe water collection needs a lot of effort and the water point is far away, they report collecting safe drinking water less often. The strongest predictors of safe water collection behavior were norms, such as others’ behavior in the household (*β* = .239) and village (*β* = .341). If respondents think that a lot of others in the household and village collect safe drinking water, they report collecting safe drinking water more often. The ability to collect enough drinking water (difficult water; *β* = −.080) is negatively associated with the target behavior. If people think they are not able to collect enough drinking water, they report collecting safe drinking water less often. Remembering was assessed in two ways: remembering “pay attention” (*β* = .102) and remembering “forgetting in the last 24 hours” (*β* = −.068). If people pay attention to performing the desired behavior, they report collecting safe drinking water more often, but if they forget about it, they report collecting less often. Communication (*β* = .110) was also positively associated with safe water collection behavior; if people communicate more about safe drinking water collection, they report collecting safe drinking water more often.

**Table 4:** Linear regression of RANAS psychosocial factors explaining the safe drinking water collection

Factor group	Behavioral factors	M (SD)	β	B
Risk factors	Vulnerability	1.96 (1.23)	.012	.016
	Severity	4.14 (.99)	−.036	−.056
	Health knowledge	9.97 (1.80)	.009	.008
Attitude factors	Belief effort	2.09 (1.66)	−.065**	−.060
	Belief time consuming	1.91 (1.51)	.054	.055
	Belief distance (far away)	2.52 (1.67)	−.114***	−.105
	Belief certain for prevent.	3.93 (1.26)	.006	.007
	Feelings	4.07 (1.22)	−.042	−.053
Norm factors	Others’ behavior househ.	3.94 (1.53)	.239***	.241
	Others’ behavior village	3.93 (1.45)	.341***	.361
	Others’ approval	4.15 (1.16)	.000	.000
	Personal obligation	4.16 (1.29)	−.045	−.054
Ability factors	Confidence in perform.	3.93 (1.33)	.052	.061
	Difficult water	1.96 (1.55)	−.080*	−.080
	Difficult time	1.72 (1.37)	−.043	−.048
	Barriers distance	3.77 (1.35)	−.032	−.037
Self-regulation factors	Coping plan	.30 (.46)	.007	.022
	Commitment (committed)	4.00 (1.21)	−.019	−.024
	Commitment (important)	4.25 (1.06)	.012	.017
	Remembering (pay attent.)	3.68 (1.36)	.102**	.115
	Remembering (forg. Last 24 h)	2.15 (1.68)	−.068**	−.062
Additional factor	Communication	3.85 (1.26)	.110***	.134

Note. *p ≤ .05, **p ≤ .01, ***p ≤ .001. Adj. *R*^*2*^ = .74.6, *N* = 621. All responses were recorded on 5-point Likert scales with choices from “1 - not at all” to “5 – very much”. Coping plan scale: 0–1 (No/Yes). Health Knowledge: sum scale (0–15)

This means that an increase in safe drinking water collection frequencies can be expected if any of these eight significant RANAS psychosocial factors increases while all other factors hold stable. An increase in water collection frequency of 0.6% can be expected in respondents who believe that water collection is not effortful and of 10.5% in those who believe that a safe well is not far away. Water collection frequency should be expected to increase by 24% in respondents who believe that water collection from a safe well is performed by many others in the household, and by 36% in those who hold the same belief about others in the village. An increase of 0.8% can be expected in respondents who think that they are able to collect enough water, of 12% in those who pay attention to collecting water from safe well, and of 0.6% in people who did not forget it. Lastly, an increase of 13% should also be expected in those who communicate more about safe drinking water. Consequently, if we target significant psychosocial factors with specific behavior change interventions we expect people to collect safe drinking water more frequently after the intervention. To identify which psychosocial factors are determinants of safe water transportation and storage behavior, we again applied multiple linear regression analysis using self-reported safe water transportation and storage behavior as outcome and RANAS psychosocial factors as predictors. Only owners of specific containers with lids for water were included in the analysis (*N =* 170). The RANAS model explained 40.9% of the variance of safe water transportation and storage frequencies (see Table 5).

**Table 5:** Linear regression of RANAS psychosocial factors explaining the water transportation and storage in specific container with lid

Factor group	Behavioral factors	M (SD)	β	B
Risk factors	Vulnerability	1.78 (1.11)	−.061	−.067
	Severity	4.25 (.94)	−.248***	−.322
	Health knowledge	10.40 (1.60)	−.036	−.027
Attitude factors	Belief effort	1.56 (1.30)	−.021	−.020
	Belief time consuming	1.56 (1.28)	.020	.019
	Belief expensive	1.29 (.80)	.026	.039
	Belief certain for prevention	4.31 (1.05)	−.136	−.159
	Feelings	4.38 (.94)	.124	.162
Norm factors	Others’ behavior househ.	4.35 (1.19)	.374***	.386
	Others’ behavior village	3.56 (1.25)	−.155*	−.152
	Others approval	4.49 (.88)	.023	.032
	Personal obligation	2.71 (1.76)	−.125	−.087
Ability factors	Confidence in performance	4.21 (1.03)	.173	.205
	Difficult time	1.21 (.70)	−.024	−.042
Self-regulation factors	Coping plan	.25 (.43)	.030	.086
	Commitment (committed)	4.32 (.97)	.076	.096
	Commitment (important)	4.31 (1.07)	−.055	−.063
	Remembering (pay attention)	4.06 (1.17)	.157	.165
	Remembering (forgetting last 24 h)	1.71 (1.28)	−.124	−.119
Additional factor	Communication	3.79 (1.23)	.176*	.175

Note. *p ≤ .05, **p ≤ .01, ***p ≤ .001. Adj. *R*^*2*^ = .409. *N* = 170. All responses were recorded on 5-point Likert scales with choices from “1 - not at all” to “5 – very much”. Coping plan scale: 0–1 (No/Yes). Health Knowledge: sum scale (0–15)

Four factors were significant predictors of safe drinking water transportation and storage in specific containers with lids: severity (i.e., the perceived severity of contracting a disease), others’ behavior household, others’ behavior village, and communication. Others’ behavior in the household (*β* = .374), that is, how many others in the household perform a target behavior, and severity (*β* = −.248), the perceived severity of contracting diarrhea, in other words the consequences for the participant’s personal and economic life, are the strongest predictors for safe drinking water transportation and storage. Additionally, communication (*β* = .176), talking to others about safe drinking water transportation and storage, is also a significant predictor of the desired behavior. A negative association between water transportation and storage and others’ behavior in the village (*β* = −.155) could be explained with a suppressor effect (i.e., a correlation with a positive and significant outcome) in a linear regression analysis.

This means that an increase in safe drinking water transportation and storage can be expected if any of these four significant RANAS psychosocial factors increase while all other factors hold stable. An increase in safe water collection and storage of 32% can be expected in respondents who perceive that contracting diarrheal disease would severely impact their lives. Further, an increase in safe water collection and storage should be expected of 39% in respondents who believe that safe water collection and storage is performed by many others in the household and of around 18% in those who communicate with others about water transportation and storage in specific containers with lids. Again, if we target significant predictors with specific behaviour change interventions, we expect people to transport and store drinking water in specific containers with lids more frequently after the intervention.

### RQ2: Is there a relationship between mental health and a) safe drinking water collection and b) water transportation and storage?

To examine our second research question, we applied a Pearson correlation. We found a significant negative relationship between mental health (SRQ-20 sum scale 0–20, cutoff ≥7; dummy variable for mental health: poor =1 and good =0) and safe drinking water collection behavior self-reported on 5-point Likert scale *p* = .01, *r* = −.104. Further statistical analysis showed that there is no statistically significant relationship between mental health and water transportation and storage.

### RQ3: Does mental health moderate a) safe drinking water collection behavior and b) water transportation and storage?

To evaluate the third research question, we applied moderation analysis using macro PROCESS for SPSS [33]. We tested moderation models for interaction (when two variables influence each others effects). All the psychosocial factors from the RANAS model that were included in regression analyses and described in the previous section were tested separately within a statistical moderations model as predictors (X). Mental health was included as moderator (M), and safe drinking water collection behavior as outcome (Y). We used bootstrapping with 10,000 samples to estimate the confidence intervals of interaction effects (interaction between mental health and psychosocial factors on water collection behavior). The levels of the moderator variable were calculated with simple slopes analysis: values for the dichotomous moderator are the two values poor = 1 versus good = 0 mental health (see Table 6).

**Table 6:** Interaction effects between mental health and RANAS psychosocial factors on self-reported safe drinking water collection behavior

Interactions of RANAS psychosocial factors with mental health	*b,* 95% CL	*t*	Conditional effects at values of mental health	
			1 = poor	0 = good
Others’ behavior village	.100* [.062, .194]	2.09	.927***	.827***
Remembering (pay attention)	.153* [.015, .291]	2.17	.749***	.596***
Remembering (forgetting last 24 h)	.178* [−.335, −.023]	−2.24	−.613***	−.435***
Commitment (important)	−.250* [−.475, −.025]	−2.18	−.316***	−.067

Notes. *p ≤ .05, **p ≤ .01, ***p ≤ .001. *N* = 634–636 (*N = 2–4* missing data), confidence intervals: 95% CL [LL, UL]. Levels of moderator calculated with simple slopes analysis: values for dichotomous moderators are the two values of the moderator. Conditional effects of X (safe drinking water collection) by Mental Health (0 = good, 1 = poor). Mental Health accessed on SRQ-20 scale (0–20), cutoff point ≥7: poor = 1, good = 0

The analysis revealed significant interaction effects between mental health and four psychosocial factors: others’ behavior village (*b = −*.100, 95% CI [.062, .194], *t =* 2.09*, p ≤* .05), remembering “pay attention” (*b =* .153, 95% CI [.015, .291], *t =* 2.17*, p ≤* .05), remembering “forgetting last 24h” (*b =* .178, 95% CI [−.335, −.023], *t = −* 2.24*, p ≤* .05), and commitment “important” (*b = −*.250, 95% CI [−.475, −.025], *t = −* 2.18*, p ≤* .05). In other words, the strength of these psychosocial factors’ influence on water collection behavior depends on the mental health condition of the respondent. The effects were significantly higher in respondents with poor mental health than in those with good mental health: Those with poor mental health are more likely to collect safe drinking water if they think that a lot of others in the village also collect safe drinking water. They also have to pay more attention to collect safe drinking water, and if they forget about it, they collect safe drinking water less often. Moderation analysis also showed that lack of commitment to collecting safe drinking water is a significant negative predictor in people with poor mental health. Commitment had no influence on safe drinking water collection in respondents with good mental health.

The moderations model that includes safe drinking water transportation and storage as outcome again used all RANAS psychosocial factors included in regression analysis as predictors and mental health as moderator (dichotomous variable: poor = 1, good =0). All the RANAS psychosocial factors were tested separately within the moderations model.

The analysis revealed significant interaction effects between mental health and three psychosocial factors (see Table 7): severity (*b =* −.496, 95% CI [−.960, −.032], *t =* − 2.11*, p ≤* .05), belief effort—the belief that transporting and storing water requires substantial effort— (*b =* .294, 95% CI [.004, .584], *t =* 2.00*, p ≤* .05), and others’ approval (*b = −*.980, 95% CI [*−* 1.458, *−*.503], *t = −* 4.05*, p ≤* .001). In other words, the influence of severity, belief effort, and others’ approval on the safe drinking water transportation and storage behavior again depends on the mental health condition of the respondent.

**Table 7:** Interaction effects between mental health and RANAS psychosocial factors on self-reported safe drinking water transportation and storage behavior

Interactions of RANAS psychosocial factors with mental health	*b*, 95% CL	*t*	Conditional effects at values of mental health	
			1 = poor	0 = good
Severity	−.496* [−.960, −.032]	−2.11	−.526*	−.031
Belief effort	.294* [.004, .584]	2.00	.141	−.154*
Others’ approval	−.980*** [−1.458, −.503]	−.4.05	−.425*	.556***

Notes. *p ≤ .05, **p ≤ .01, ***p ≤ .001. *N* = 172, confidence intervals: 95% CL [LL, UL]. Levels of moderator calculated with simple slopes analysis: values for dichotomous moderators are the two values of the moderator. Conditional effects of X (safe drinking water transportation and storage behavior) by mental health (0 = good, 1 = poor). Mental health accessed on SRQ-20 scale (0–20), cutoff point ≥7: people with a score equal or above 7, poor = 1, people with a score below 7, good = 0 mental health

The perceived severity of contracting diarrhea was a significant negative predictor of water transportation and storage in people with poor mental health, but not in people with good mental health. That is, people with poor mental health perceive stronger negative consequences of contracting diarrhea for personal and economic situation, and they collect safe drinking water more often. In contrast, the belief that transporting and storing water requires substantial effort had no influence on behavior in people with poor mental health, but in people with good mental health it was a significant negative predictor. That is, people collect safe drinking water more often when they think that water collection does not require a lot of effort. The influence of others’ approval on the safe drinking water transportation and storage of respondents with poor mental health was negative; it was also significantly lower than on those with good mental health, on whom the influence was significant and positive: The influence of others’ approval was interrupted by poor mental health.

### RQ4: Are there differences between individuals with good and poor mental health in RANAS psychosocial factors affecting a) safe drinking water collection behavior and b) water transportation and storage?

**Fig. 2:**
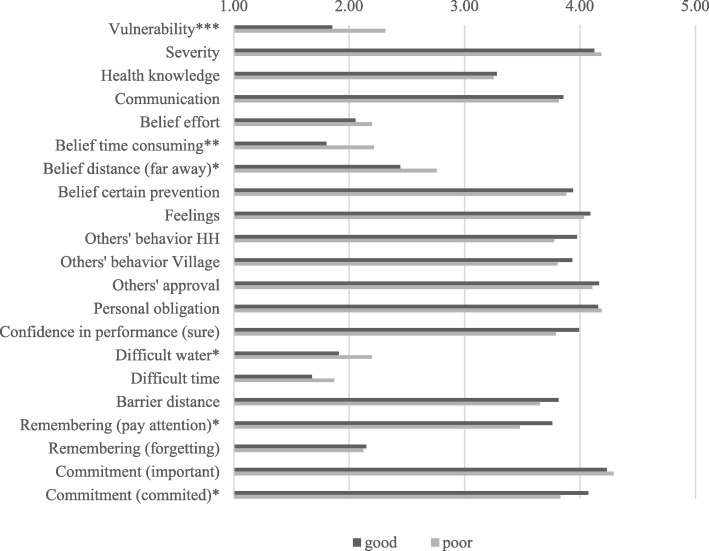
ANOVA mean comparison of RANAS psychosocial factors explaining safe drinking water collection behavior by Mental Health. Note. *p ≤ .05, **p ≤ .01, ***p ≤ .001. *N* = 638. All questions included 5-point Likert scales and response choices from “1 - not at all” to “5 – very much”. Health Knowledge: sum scale (0–15). Mental Health on SRQ-20 scale, cutoff point ≥7: poor = 1, good =0

To answer our final research question, we applied ANOVA mean comparison analysis. We included all RANAS psychosocial factors explaining safe drinking water collection by mental health condition to compare the means of the two groups. Significant differences between the two groups were found in six RANAS psychosocial factors: Vulnerability, belief time consuming, belief distance (far away), difficult water, remembering (pay attention), and commitment (committed) (see Fig. 2). People with poor mental health feel more vulnerable than people with good mental health to contracting a disease if they do not collect safe drinking water. They also believe more strongly than people with good mental health that safe drinking water collection needs more time, that the water collection point is far away, and that it is difficult to collect enough drinking water. According to our analysis results, individuals with poor mental health also pay less attention to collecting drinking safe water more often, and are less committed to collecting drinking safe water.

Further analysis revealed no statistically significant differences between respondents with good mental health and those with poor mental health regarding RANAS psychosocial factors explaining water transportation and storage behavior.

## Discussion

### Interpretation of results

This study investigated direct and indirect links between mental health and safe drinking water collection, transportation, and storage. The aim of our study was to design evidence-based behavior change interventions for a vulnerable population in rural Malawi that address not only people’s behavior but also their mental health condition.

Almost a third of the study respondents in a population of rural Malawi exhibited poor mental health, which is in line with other studies from Malawi [14, 15]. The respondents with poor mental health in Kapelula can be characterized as experiencing more hunger, suffering more from diarrhea, and being more anxious about the future situation of the family. They are also significantly less likely to be literate or married than are people with good mental health.

First, RQ1 (Which psychosocial factors are behavioral determinants a) for safe drinking water collection and b) for water transportation and storage?), was answered using the RANAS approach to detect the psychosocial factors that influence safe drinking water collection, transportation and storage behaviors in all respondents included in our study irrespective of their mental health condition.

Results showed that people report collecting safe water more often the less they perceive it as effortful, distant, and difficult and the less that they forget to execute the behavior. However, they report performing the behavior more often if they perceive that others in the household and the village also perform the behavior, the more they pay attention to remembering it, and the more they talk about it. Safe transportation and storage is performed more the more others perform it in the household, and the more they talk about it. How well does the RANAS model explain the behaviors? The RANAS model explained 74.6% of variance in the collection behavior, but less in the transportation and storage behaviors (40.9%). Our study results confirmed that the RANAS model predicts safe drinking water behaviors very accurately, in line with previous research [8, 25, 26] and as shown in a review of 14 studies in 10 countries [27].

Second, our study results for RQ2 (Is there a relationship between mental health and a) safe drinking water collection and b) water transportation and storage?), showed a negative association between poor mental health and self-reported safe drinking water collection behavior, in line with our assumptions. However, contrary to our expectations, we could not find an association between poor mental health and water transportation and storage behaviors. It could be that the use of a safe container changes if the household already has such a container with a lid.

Third, results for RQ3 (Does mental health moderate a) safe drinking water collection behavior and b) water transportation and storage?) confirmed that the influence of several psychosocial factors on the safe water behaviors was moderated by mental health.

The effects of other people’s behavior in the village, paying attention, and forgetting on collection behavior depend on mental health condition. The influence of these factors on people with poor mental health was higher than their influence on people with good mental health. Being less committed to the behavior only influenced respondents with poor mental health, and high rates of commitment in people with poor mental health actually decreased their performance of water collection behavior. It could be that the pressure exerted by commitment resulted in provoked reactance in people with poor mental health. Reactance leads to behavioral, affective, and cognitive effects and is well known from psychological reactance theory [34, 35]. Consequently, interventions for vulnerable people with poor mental health using behavior change techniques that target commitment should be implemented together with or after interventions on mental health.

In summary, these findings suggest that behavior change interventions targeting two psychosocial factors—others’ behavior in a village and remembering (‘pay attention’ and ‘forgetting last 24h’)—would increase frequency of safe drinking water collection both in those with poor mental health and those with good. However, behavior change interventions focusing on commitment would decrease the frequency of safe drinking water collection in those with poor mental health.

Safe water transportation and storage behavior was not directly affected by mental health. However, further moderation analysis revealed that the perception of contracting a disease (severity) only influenced people with poor mental health; it had no influence on people with good mental health. Perceived effort was impaired by poor mental health and influenced water transportation and storage only in people with good mental health. The effect of others’ approval on transporting and storing water was also impaired by poor mental health and worked only in people with good mental health. In contrast, high rates in others’ approval decreased water transportation and storage behavior in people with poor mental health. All our respondents already owned specific containers with lids, so the reaction of people with poor mental health to the approval of others might again be explained by reactance [34, 35].

These findings suggest that behavior change interventions focusing on severity would increase safe drinking water transportation and storage in specific containers with lids only in people with poor mental health. However, behavior change interventions targeting belief effort and others’ approval would increase safe drinking water transportation and storage only in people with good mental health. Consequently, behavior change interventions that target others’ approval should be implemented together with or after interventions on mental health for vulnerable people with poor mental health.

Fourth, results for RQ4 (Are there differences between individuals with good and poor mental health in RANAS psychosocial factors affecting a) safe drinking water collection behavior and b) water transportation and storage?), showed significant differences between individuals with good and poor mental health in RANAS psychosocial factors relevant for safe drinking water collection behavior. According to our findings, people with poor mental health perceive themselves to be more vulnerable to contracting diarrhea or other diseases; they also feel that safe drinking water collection needs effort, is time consuming and that it is thus difficult to obtain enough water for daily needs. Concentration on daily tasks, paying attention, and collecting safe drinking water is also lower in people with poor mental health than in people with good. These results are not surprising; they may well mirror such symptoms of depression and anxiety as higher vulnerability, tiredness, absence of confidence in performance, and lack of concentration in people with poor mental health. However, contrary to our expectations, no significant differences emerged between the two groups in RANAS psychosocial factors related to water transportation and storage behavior. As mentioned above, it seems to be easy to perform and easy to remember even for people with poor mental health.

In summary, our study results confirmed the direct link (e.g. negative association) between mental health and safe drinking water collection, but not between transportation and storage. The results also confirmed the indirect link, the interaction effects of mental health condition with some psychosocial factors influencing safe water collection and safe water transportation and storage in specific containers. The analysis revealed that individuals with poor mental health feel more vulnerable, seem to experience challenges with distance, time, and difficulty of fetching water from a safe well and with remembering to perform the behavior daily. They also experience more hunger and diarrhea and are more anxious about the future situation of their families than people with good mental health. These results are not surprising, as reduced mental health is frequently accompanied by anxiety, decreased energy, depressed mood, and depressed thoughts [36]. The results of this study are in line with our previous findings from rural Malawi about characteristics such as marital status and literacy of the last non-owners of latrines [37]. The results of this study also support our previous findings in Zimbabwe that depression has a negative influence on daily hygienic activities such as hand washing and impairs the influence of psychosocial factors on hand washing with soap in primary school children [22].

## Conclusions

The study findings are important for policy makers and NGOs for several reasons. First, we strongly recommend including mental health measurements (e.g. SRQ-20) in surveys addressing behavior change in safe drinking water collection, transportation, and storage in specific containers with lids. Second, vulnerable people with poor mental health should receive interventions targeting mental health before or concurrently with interventions targeting behavior change in water collection, transportation, and storage. Next, if treatment for poor mental health is not possible for any reason, our study results can be used to decide which interventions should be implemented with the whole population and which should be tailored to those with poor mental health. E.g. Behavior change techniques addressing safe water collection should target the perception of others’ behavior in the household and communication irrespective of the mental health condition of the target population. In line with the RANAS catalog of behavior change techniques [24], the first strategy is social influence or persuasion, which focuses on others’ behavior in the household: Inform people about others’ behavior and encourage people to commit to safe water collection and make their commitment public. The commitment can be given orally in front of an audience or in writing at a public place. The second strategy is communication: Prompt talking to others about safe water collection and invite participants to talk to others about collecting drinking water from a safe well. For both behavior change strategies, we suggest community meetings as communication channel.

The present study results imply that populations with a significant proportion of individuals with poor mental health will benefit from interventions that mitigate mental health before or concurrently with behavior change interventions for WASH. There is evidence that specific population-level interventions have a positive effect on mental well-being, and they have been successfully applied at scale in refugee camp populations in Africa; examples include e.g. narrative exposure therapy (NET) [38, 39] and, in other African settings, group based interpersonal therapy (IPT-G) for treatment of depression and anxiety [40, 41]. This research is especially relevant in emergency contexts, as it indicates that mental health measures will make any WASH interventions more effective if implemented before or concurrently with them.

The present study is a cross-sectional study and further research is needed to confirm our results to determine whether interventions to increase mental health should be implemented before or concurrently with WASH interventions.

## Data Availability

The dataset generated and analyzed during the current study is available in the [https://figshare.com/s/dd9a99c673a65aa97092] repository, [DOI: 10.6084/m9.figshare.6040625].

## Abbreviations

BCT: Behavior change technique
IPT-G: Group based interpersonal therapy
MRCS: Malawi Red Cross Society
NET: Narrative exposure therapy
NGO: Nongovernment organizations
RANAS: Risks, attitudes, norms, abilities, and self-regulation
SRQ-20: Self-Reporting Questionnaire
UN: United Nations
UNICEF: United Nations Children’s Fund
WASH: Water, sanitation, and hygiene
WHO: World Health Organisation
